# The non‐peptidomimetic IAP antagonist ASTX660 sensitizes colorectal cancer cells for extrinsic apoptosis

**DOI:** 10.1002/2211-5463.13096

**Published:** 2021-02-19

**Authors:** Gertrud Knoll, Martin Ehrenschwender

**Affiliations:** ^1^ Institute of Clinical Microbiology and Hygiene University Hospital Regensburg Germany

**Keywords:** apoptosis, ASTX660, SMAC mimetic, TNF, TRAIL

## Abstract

Apoptosis resistance worsens treatment response in cancer and is associated with poor prognosis. Inhibition of anti‐apoptotic proteins can restore cell death and improve treatment efficacy. cIAP1, cIAP2, and XIAP belong to the inhibitor of apoptosis protein (IAP) family and block apoptosis. Targeting IAPs with peptides or peptidomimetics mimicking the IAP‐antagonizing activity of the cell's endogenous IAP antagonist SMAC (SMAC mimetics) showed promising results and fueled development of novel compounds. ASTX660 belongs to the recently introduced class of non‐peptidomimetic IAP antagonists and successfully completed phase I clinical trials. However, ASTX660 has thus far only been evaluated in few cancer entities. Here, we demonstrate that ASTX660 has cell death‐promoting activity in colorectal cancer and provide a head‐to‐head comparison with birinapant, the clinically most advanced peptidomimetic IAP antagonist. ASTX660 facilitates activation of the extrinsic apoptosis pathway upon stimulation with the death ligands TNF and TRAIL and boosts effector caspase activation and subsequent apoptosis. Mechanistically, ASTX660 enhances amplification of death receptor‐generated apoptotic signals in a mitochondria‐dependent manner. Failure to activate the mitochondria‐associated (intrinsic) apoptosis pathway attenuated the apoptosis‐promoting effect of ASTX660. Further clinical studies are warranted to highlight the therapeutic potential of ASTX660 in colorectal cancer.

## Graphical abstract

Inhibitor of apoptosis proteins (IAPs) contribute to apoptosis resistance in cancer, a main cause of treatment failure. We demonstrate that the novel non‐peptidomimetic IAP antagonist ASTX660 promotes cell death in colorectal cancer. ASTX660 facilitates activation of the extrinsic apoptosis pathway upon stimulation with the death ligands TNF and TRAIL, boosts effector caspase activation, and enhances subsequent apoptosis.

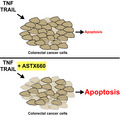

**Abbreviations**
BAK: BCL2 antagonist/killer
BAX: BCL2‐associated X protein
BCL‐2: B‐cell lymphoma 2
BID: BH3‐interacting domain death agonist
BIM: BCL‐2‐interacting mediator of cell death
BIR: baculoviral IAP repeat
CRC: colorectal cancer
IAP: inhibitor of apoptosis protein
SM: SMAC mimetic
SMAC: second mitochondria‐derived activator of caspase
TNF: tumor necrosis factor
TRAIL: tumor necrosis factor‐related ligand
TWEAK: TNF‐related weak inducer of apoptosis
XIAP: X‐linked inhibitor of apoptosis

Cancer cells commonly fail to undergo apoptosis, a tightly regulated cellular suicide program [1]. Apoptosis resistance promotes cancer growth and is associated with poor treatment response. Characterization of proteins involved in apoptosis regulation highlighted members of the inhibitor of apoptosis (IAP) protein family as novel therapeutic targets. In transformed cells, three IAP proteins effectively block apoptosis: cellular IAP1 (cIAP1), cellular IAP2 (cIAP2), and X‐chromosome‐linked IAP (XIAP) [2, 3, 4]. The decisive role of IAP proteins spurred development of small molecules with IAP‐antagonizing activity. The cell's endogenous IAP antagonist second mitochondria‐derived activator of caspases (SMAC) served as model and yielded a first generation of SMAC‐based peptides and peptidomimetics mimicking SMAC's minimal N‐terminal IAP binding motif AVPI. Later on, conformation‐constrained bicyclic lactam structures imitating the geometry of the AVPI peptide were developed, with either one (monovalent) or two (bivalent) AVPI‐like binding motifs [5]. Among these bivalent third‐generation SMAC mimetics (SM), birinapant (previously TL32711) is most advanced in clinical trials [6, 7]. Recently, a fragment‐based screening approach identified non‐peptidic compounds with millimolar IAP affinity and resulted in development of ASTX660, a non‐peptidomimetic antagonist of cIAP1/2 and XIAP [8, 9, 10]. In contrast to birinapant, ASTX660 is orally bioavailable [11]. Preclinical data indicated synergistic effects for this novel IAP antagonist in combinatorial treatments in head and neck cancers and acute myeloid leukemia [12, 13, 14, 15]. However, ASTX660 has thus far not been evaluated in other cancer entities.

Here, we show that in colorectal cancer (CRC) ASTX660 itself is not cytotoxic but efficiently depletes cIAPs. ASTX660 facilitates caspase activation upon death receptor stimulation and sensitizes CRC cells to TRAIL‐ and TNF‐induced apoptotic cell death. Notably, the bivalent IAP antagonist birinapant more efficiently primed CRC cells for apoptosis than ASTX660 did. Failure to activate upstream (initiator) caspases in the apoptotic cascade or loss of mitochondria‐mediated amplification of the death signal strikingly attenuated the apoptosis‐promoting effect of ASTX660. Further clinical studies are warranted to elucidate the therapeutic potential of ASTX660 in CRC.

## Materials and methods

### Cell lines, antibodies, and reagents

Colo205, HCT8, HCT116, HT29, LoVo, LS174T, RKO, SW480, and SW948 cells were obtained from the German Collection of Microorganisms and Cell Culture (DSMZ, Braunschweig, Germany). HCT116 BAX/BAK DKO cells were kindly provided by Richard Youle (National Institutes of Health, Bethesda, MD, USA) [16]. BID‐deficient HCT116 cells and the complemented variants thereof (HCT116 BID KO+BID D60E, HCT116 BID KO+BID G94E) were a kind gift from Xu Luo (University of Nebraska Medical Center, Nebraska, USA) [17]. HCT116 BIM KO and HCT116 caspase‐8 KO cells were provided by Hamsa Puthalakath (La Trobe University, Bundoora, Australia) [18]. SW48 and DLD1 cells and BAX/BAK‐deficient variants thereof were purchased from Sigma (Steinheim, Germany). All cell lines were maintained in RPMI 1640 medium (PAN Biotech, Aidenbach, Germany) with 10% (v/v) fetal calf serum (Sigma). Antibodies: caspase‐3 (#9662), cIAP1 (#9770): Cell Signaling (Beverly, MA, USA); tubulin (#MS‐581): Dunnlab (Asbach, Germany); XIAP (#610716): BD Biosciences, Heidelberg, Germany; cIAP2 (#ab23423): Abcam (Cambridge, UK); Chemicals: MTT (3‐[4,5‐dimethylthiazol‐2‐yl]‐2,5‐diphenyl tetrazolium bromide): Biomol, (Hamburg, Germany); ASTX660, birinapant, QVD‐OPh: Hycultec (Beutelsbach, Germany). KillerTRAIL was purchased from Apronex (Jesenice u Prahy, Czech Republic). TNF was a kind gift from Daniela Maennel (University of Regensburg, Germany).

### MTT‐based cell viability assay

Cells were seeded in 96‐well plates (2 × 10^4^ cells/well) and challenged with the indicated concentrations of the indicated substances in duplicates (technical replicates). Unless indicated otherwise, cell viability was determined 18 h after stimulation using MTT staining (2 h at 37 °C). Staining intensity was measured at 595 nm, and the mean was calculated from the technical replicates of each experiment. The mean value for untreated controls was set to 100%. For any other condition, the MTT staining intensity is given relative to the corresponding untreated group (percent of control). Mean values were calculated from three independent experiments and graphically presented as heatmaps. The corresponding numeric values ± SD can be found in Tables S1–S7.

### Western blot analysis

Western blot analysis was essentially performed as described previously [19]. In brief, cells were harvested by centrifugation and lysed in 4× Laemmli sample buffer [8% (w/v) SDS, 0.1 m dithiothreitol, 40% (v/v) glycerol, 0.2 m Tris, pH 8.0] supplemented with phosphatase inhibitor cocktails I and II (Sigma). Samples were sonicated and boiled for 5 min at 96 °C before proteins were separated by SDS/PAGE and transferred to PVDF membranes. To block nonspecific binding sites, membranes were incubated in TBS containing 0.1% (v/v) Tween 20 and 5% (w/v) dry milk before primary antibodies of the specificity of interest were added. Antigen–antibody complexes were visualized using horseradish peroxidase‐conjugated secondary antibodies (Dako, Hamburg, Germany) and ECL technology (Pierce, Rockford, IL, USA).

### Flow cytometry

Cell death was assessed by annexin‐V and 7‐aminoactinomycin D (7‐AAD) staining. In brief, HCT116 and SW48 cells were challenged with the indicated concentrations of KillerTRAIL, ASTX660 or both for 6 h or left untreated. Afterward, cells were stained with 7‐AAD and annexin‐V (4 °C for 15 min in the dark). A FACSCanto flow cytometer (BD Biosciences) was used for analysis following standard procedures [20].

### Caspase activity assay

Fluorescence‐based assessment of caspase activity has also been described in our previous studies [21, 22]. Caspase activity was measured using the caspase‐3/‐7 activity kit (AAT Bioquest, Sunnyvale, CA, USA) according to manufacturer's instructions. Emitted fluorescence was quantified using a Victor3 Multilabel Reader (Perkin Elmer, Waltham, MA, USA).

## Results

### ASTX660 depletes cIAP2 and sensitizes CRC cells to death receptor‐mediated cytotoxicity

**Fig. 1:**
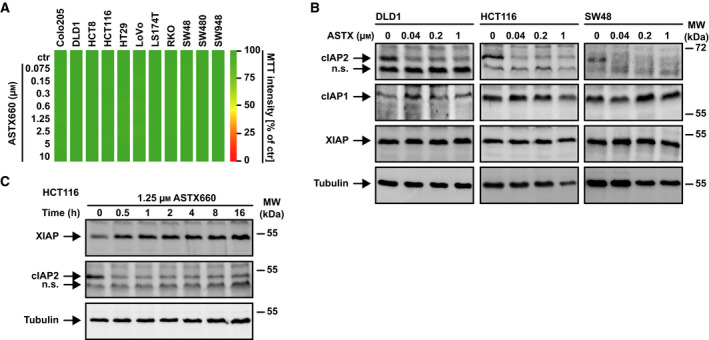
ASTX660 depletes cIAP2 in CRC cells. (A) Colorectal cancer cell lines were challenged with the indicated concentrations of ASTX660, and cell viability was measured after 24 h. Numeric values ± SD can be found in Table S1. (B) Cells were challenged with the indicated concentrations of ASTX660 for 24 h. After washing and cell lysis, western blot analyses were performed with antibodies specific for the indicated proteins. Detection of tubulin served as a loading control. (C) HCT116 cells were challenged with ASTX660 (1.25 µm) for the indicated periods. For (A), data shown are mean values from three independent experiments; for (B) and (C), data shown are representative of at least two independent experiments performed.

**Fig. 2:**
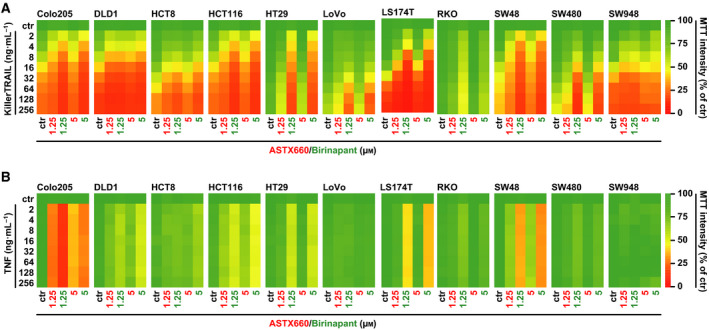
ASTX660 sensitizes CRC cells to TRAIL and TNF. (A, B) Cells were challenged with the indicated concentrations of KillerTRAIL or TNF in the presence and absence of ASTX660 or birinapant (1.25 and 5 µm). Numeric values ± SD can be found in Tables S2 and S3. For (A) and (B), data shown are mean values from three independent experiments.

**Fig. 3:**
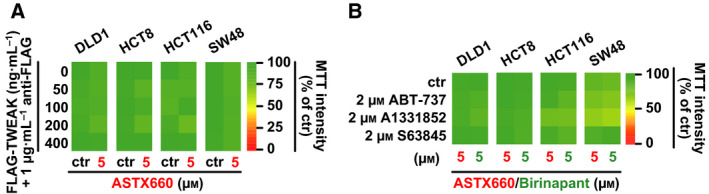
ASTX660 does not enhance cytotoxicity of the non‐death ligand TWEAK or antagonists of BCL‐2 family proteins. (A) Cells were challenged with the indicated concentrations of FLAG‐TWEAK for 24 h in the presence and absence of ASTX660 (5 µm). Anti‐FLAG antibody was added (1 µg·mL^−1^) to enhance bioactivity of the ligand [49]. (B) Cells were challenged with the indicated concentrations of ABT‐737 (targets BCL‐2, BCL‐XL, and BCL‐W), A1331852 (targets BCL‐XL), or S63845 (targets MCL‐1) in the presence of ASTX660 or birinapant (5 µm). Numeric values ± SD can be found in Tables S6 and S7. For (A and B), data shown are mean values from three independent experiments.

Treatment with ASTX660 alone displayed no cytotoxic effects in various CRC cell lines in doses up to 10 µm (Fig. 1A and Fig. S1), whereas ASTX660 concentrations as low as 40 nm were sufficient to decrease cIAP2 (but not cIAP1) levels rapidly (Fig. 1B,C). SM rarely induce XIAP degradation, and XIAP levels indeed remained unaffected in ASTX660‐treated cells (Fig. 1B,C). Combination of ASTX660 with an oligomerized, highly bioactive TRAIL variant (KillerTRAIL, hereafter referred to as TRAIL) enhanced death receptor‐mediated cytotoxicity in all CRC cell lines tested (Fig. 2A). Interestingly, the TRAIL‐sensitizing effect of ASTX660 appeared to be less pronounced than that of the bivalent SMAC mimetic birinapant. Increasing ASTX660 concentrations did not overcome this difference and thus rendered a dose‐dependent phenomenon unlikely (Fig. 2A). ASTX660 and birinapant also sensitized CRC cell lines toward TNF, a death ligand with (compared to TRAIL) lower cytotoxic potential (Fig. 2B). Again, TNF/birinapant treatment was superior to TNF/ASTX660 in terms of cell death induction, essentially confirming the higher efficacy of birinapant to boost death receptor‐mediated killing in CRC cells (Fig. 2B). In contrast to TRAIL, presence of ASTX660 or birinapant was not sufficient to elicit TNF‐induced cytotoxicity in all cell lines tested (Fig. 2B). LoVo and SW948 cells, for example, displayed no or only marginal loss of viability upon challenge with ASTX660/TNF and birinapant/TNF (Fig. 2B). The failure of TNF to exceed the threshold for cell death induction could (among others) reflect the relatively low cytotoxic potential of this ligand or be attributable to low/no expression levels of its cognate death receptor, TNF‐receptor 1. Notably, ASTX660 did not sensitize CRC cells to TWEAK, a non‐death ligand of the TNF superfamily (Fig. 3A). Likewise, ASTX660 did not enhance intrinsic apoptosis induced by inhibition of BCL‐2 family proteins (‘BH3 mimetics’) (Fig. 3B). Collectively, our data confirmed that in CRC cells ASTX660 (a) displayed no cytotoxicity as single agent, (b) acted as an antagonist of cIAP2, and (c) enhanced death receptor‐mediated cell death.

### Death receptor‐mediated apoptosis is enhanced by ASTX660

**Fig. 4:**
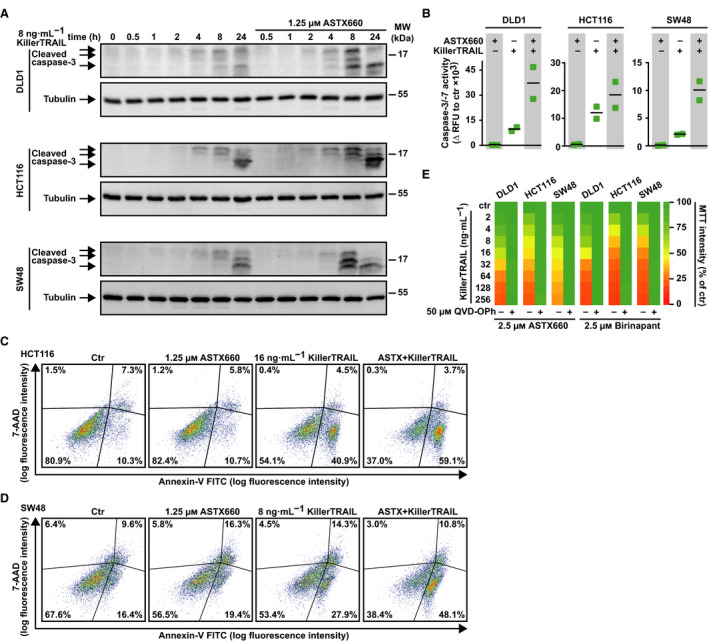
TRAIL‐induced apoptosis in CRC is boosted by the IAP antagonist ASTX660. (A) Cells were challenged with KillerTRAIL (8 ng·mL^−1^) for the indicated periods. After washing and cell lysis, western blot analyses were performed with antibodies specific for the indicated proteins. Detection of tubulin served as a loading control. (B) Cells were challenged with KillerTRAIL (8 ng·mL^−1^) and/or ASTX660 (1.25 µm) for 18 h. Caspase‐3/‐7 activity was assessed using the fluorogenic substrate (DEVD)_2_‐R110. (C, D) Cells were challenged with the indicated concentrations of KillerTRAIL and/or ASTX660 for 6 h and subsequently analyzed by flow cytometry for 7‐AAD‐ and annexin‐V‐positivity. (E) Cells were challenged with the indicated concentrations of KillerTRAIL for 24 h in the presence and absence of the pan‐caspase inhibitor QVD‐OPh (50 µm). Numeric values ± SD can be found in Table S4. For (A, C, and D), data shown are representative of two experiments performed; for (B), individual data points and mean from two experiments are shown; and for (E), data shown are mean values from three independent experiments.

We next assessed the mechanisms underlying ASTX660‐granted sensitization of CRC cells to death receptor cytotoxicity. ASTX660 enhanced proteolytic processing of caspase‐3 in TRAIL‐exposed cells and likewise increased TRAIL‐induced activity of caspases 3 and 7, two critical effector caspases in the execution phase of the apoptotic cascade (Fig. 4A,B). Additionally, combinatorial treatment with ASTX660 and TRAIL yielded a higher percentage of 7‐AAD/Annexin‐V‐positive cells compared to TRAIL alone (Fig. 4C,D). Activation of caspases was essential for ASTX660/TRAIL‐mediated cytotoxicity as the pan‐caspase inhibitor QVD‐OPh provided full‐blown protection (Fig. 4E). Essentially, enhanced TRAIL cytotoxicity in the presence of ASTX660 was paralleled by an increase in signs characteristic for ongoing apoptotic cell death. Consequently, we concluded that ASTX660 boosts TRAIL‐induced activation of the extrinsic apoptosis pathway in CRC cells.

### ASTX660‐granted TRAIL cytotoxicity requires mitochondria‐mediated amplification of the death signal

**Fig. 5:**
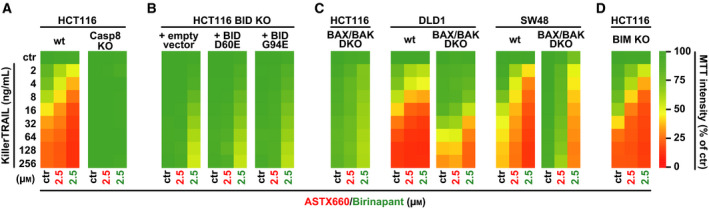
Loss BID or the pore‐forming proteins BAX/BAK attenuates ASTX660‐granted TRAIL sensitization. (A–D) Cells were challenged with the indicated concentrations of KillerTRAIL for 24 h in the presence and absence of ASTX660 and birinapant (2.5 µm). Numeric values ± SD can be found in Table S5. Data shown are mean values from three independent experiments.

Death receptor‐induced apoptosis can proceed straightforward: activation of initiator caspases (e.g., caspase 8) at the death receptor‐associated signaling complex, sufficient activation of downstream effector caspases (such as caspase 3), and finally death (so‐called type I mode). ‘Weak’ apoptosis‐promoting signals from death receptors, however, require amplification via a mitochondria‐associated pathway to trigger cell death (type II mode). In this case, caspase‐8‐mediated cleavage of the BH3‐interacting domain death agonist (BID) enables BCL‐2‐associated X protein (BAX) and BCL‐2‐antagonist/killer (BAK) to permeabilize the outer mitochondrial membrane [23, 24]. Release of apoptogenic factors fuels effector caspase activity, finally exceeding the threshold for cell death induction. Genetic loss of caspase‐8 expectedly attenuated the TRAIL‐sensitizing effect of ASTX660 and birinapant (Fig. 5A). Likewise, lack of BID and complementing BID‐deficient cells with caspase‐8‐resistant (BID D60E) or BH3‐defective (BID G94E) BID mutants reduced cytotoxicity of TRAIL/ASTX660 treatment (Fig. 5B). Finally, deletion of the pore‐forming proteins BAX and BAK rescued HCT116 cells from TRAIL/ASTX660‐induced apoptosis (Fig. 5C). Alterations in the aforementioned apoptosis‐promoting BCL‐2 family proteins also impaired cytotoxicity of TRAIL in the presence of birinapant, although to a lesser extent (Fig. 5B,C). Notably, the directly BAX/BAK‐activating pro‐apoptotic BCL‐2 protein BIM had apparently no critical role in TRAIL/SM‐induced apoptosis. In the absence of BIM, ASTX660 and birinapant both enhanced TRAIL‐induced cell death (Fig. 5D). Collectively, our data suggest that ASTX660‐mediated TRAIL sensitization relies on amplification of the death signal via the mitochondrial pathway. Hence, ASTX660 does not convert extrinsic apoptosis in CRC cells from a mitochondria‐dependent type II mode into a mitochondria‐independent type I mode.

## Discussion

Ongoing phase I/II clinical trials indicate that SM are well tolerated and have a manageable safety profile. Most SM display a rather low efficacy in cancer therapy as single agents [25]. Nevertheless, inhibition of tumor growth has been reported for ASTX660 *in vivo* and clinical studies currently evaluate efficacy in lymphoma and solid tumors [11, 26, 27]. The anti‐tumor effect of ‘SM‐only treatments’ depends on the ability of cancer cells to produce TNF and exert a cytotoxic response to this death ligand. Consequently, failure to release TNF or impaired cytotoxic TNF signaling renders cancer cells resistant to SM [28, 29, 30, 31]. Combinatorial treatment strategies with SM and exogenously added TNF (or other death‐inducing ligands from the TNF super family such as TRAIL) overcome SM resistance in various cancer entities [32, 33, 34, 35]. In line with this, our data demonstrate an ASTX660/birinapant‐mediated increase in TRAIL‐ and TNF‐induced cytotoxicity (Fig. 2A,B). Severe side effects unfortunately preclude systemic administration of TNF in therapeutically relevant doses [36]. In contrast to the good safety profile of SM, TNF production and release need to be spatially constrained. Locally restricted TNF induction occurs during an enforced cellular response to increasing osmotic pressure in the tumor microenvironment, for example [37]. Likewise, activation of cytotoxic lymphocytes in cancer immunotherapy triggers TNF release [38]. In both cases, SM sensitize cancer cells to TNF‐dependent killing. Besides TNF, lymphocyte‐mediated tumor cell apoptosis also involves cytotoxic TRAIL signaling [39]. High expression levels of IAP contribute to TNF and TRAIL resistance in cancer cells and consequently attenuate the impact of cancer immunotherapy [39, 40, 41]. Combining cancer immunotherapy with the IAP‐antagonizing activity of SM to enhance TNF‐ and TRAIL‐mediated cancer cell killing via cytotoxic lymphocytes is therefore a reasonable therapeutic approach. Compared to TNF, the cytotoxic potential of TRAIL is higher due differences in the signaling pathways predominantly activated (TNF: pro‐inflammatory vs TRAIL: pro‐apoptotic). This is reflected by our data: ASTX660/birinapant plus TRAIL is superior to ASTX660/birinapant plus TNF in cancer cell killing (Fig. 2A). TRAIL demonstrated tremendous promise in preclinical *in vitro* and *in vivo* studies (e.g., selective apoptosis induction in malignant cells) [42, 43] but clinical efficacy was initially disappointing [41]. Meanwhile, a more potent ‘second‐generation’ of TRAIL and TRAIL‐receptor agonists is evaluated in clinical trials and could perspectively be used in combinatorial treatments with SM [41].

Interestingly, the clinically advanced bivalent SM birinapant and ASTX660 differed in their efficacy to sensitize CRC cells for extrinsic apoptosis. Birinapant enhanced TRAIL‐ and TNF‐induced apoptosis more than ASTX660 did (Fig. 2A,B). In HT29, LS174T, and SW480 cells, TNF treatment displayed cytotoxic effects in the presence of birinapant, but not ASTX660 (Fig. 2B). This could reflect cell line‐specific differences in the contribution of baculoviral IAP repeat (BIR) domains to apoptosis resistance. cIAP1, cIAP2, and XIAP each contain three BIR domains, of which BIR2 and BIR3 are especially important to fulfill the anti‐apoptotic function. Mechanistically, the BIR3 domain binds to caspase‐9, restricts its activation, and thereby controls (a) initiation of the intrinsic apoptosis pathway and (b) mitochondria‐mediated amplification of the apoptotic signal generated by death receptors. The latter is especially important for the type II mode of extrinsic apoptosis. Monovalent AVPI‐based SM preferentially target BIR3. This is also claimed for ASTX660, but comparative data on ASTX660‐BIR2 affinity have not been released [8]. Birinapant and SMAC, the endogenous antagonist of IAPs, are bivalent [44]. This allows dual targeting of BIR3 and BIR2. The BIR2 domain of XIAP holds a critical role in controlling activation of the effector caspases 3 and 7 and functionally discriminates between type I and type II apoptosis upon death receptor activation [45, 46]. Hence, targeting XIAP's BIR2 domain facilitates type I apoptosis by robust activation of initiator and effector caspases and reduces dependency on BAX/BAK‐mediated amplification of the death receptor‐generated apoptotic signal [47, 48]. Not surprisingly, combination of TRAIL with the dually BIR2/BIR3‐targeting SM birinapant was more effective in BAX/BAK‐deficient cell lines than TRAIL combined with the BIR3‐binding SM ASTX660 (Fig. 5C) [11]. Likewise, ASTX660/TRAIL cytotoxicity was more severely affected by deficiency or dysfunction of BID (the ‘linker’ of death receptor‐generated apoptotic signals to the mitochondrial amplification loop) than birinapant/TRAIL cytotoxicity (Fig. 5B). A cell's individual dependency on IAP proteins and especially the anti‐apoptotic function of the BIR2 and/or BIR3 domains finally determines efficacy of birinapant and ASTX660 to unlock the extrinsic apoptosis pathway when encountering a rather weak (TNF) or strong (TRAIL) apoptotic stimulus.

## Conclusion

Collectively, our data suggest that increased TRAIL‐induced apoptosis in ASTX660‐treated cells is attributable to intensified amplification of the death signal via the mitochondrial pathway. From a therapeutic perspective, currently available clinical data of ASTX660 are encouraging: A phase I study in adults with advanced cancers or lymphoma demonstrated a manageable safety profile of ASTX660; phase II studies are currently ongoing [27]. Our data suggest that ASTX660 might also be effective in colorectal cancer although further clinical studies are certainly warranted. From the lessons learned thus far, however, SM‐based treatment regiments are unlikely to work efficiently in all patients. Identification and validation of biomarkers that reliably predict treatment response are needed to optimize individual treatment strategies and maximize treatment success. With their good safety profile, little off‐target activity and their potential to synergize with ‘traditional’ (e.g., radio‐ or chemotherapy) and ‘novel’ (e.g., immunotherapy) treatments, SM are promising compounds for future cancer treatments.

## Conflict of interest

The authors declare no conflict of interest.

## Author contributions

ME and GK designed the experiments, performed the experiments, and analyzed data. ME wrote the paper.

## Supporting information


**Fig. S1.** Absorbance spectrum of ASTX660.

**Table S1.** Numeric values ± SD to Fig. 1A.
**Table S2.** Numeric values ± SD to Fig. 2A.
**Table S3.** Numeric values ± SD to Fig. 2B.
**Table S4.** Numeric values ± SD to Fig. 4E.
**Table S5.** Numeric values ± SD to Fig. 5A‐D.
**Table S6.** Numeric values ± SD to Fig. 3A.
**Table S7.** Numeric values ± SD to Fig. 3B.

## Data Availability

The data and material used in the present study are available from the corresponding author on reasonable request.
