# Supplementary material for: The non‐peptidomimetic IAP antagonist ASTX660 sensitizes colorectal cancer cells for extrinsic apoptosis
Source: FEBS Open Bio. 2021 Feb 19;11(3):714–23. doi: 10.1002/2211-5463.13096 (PMC7931242; doi:10.1002/2211-5463.13096)
Supplement: Supplementary file 1 — Fig. S1. Absorbance spectrum of ASTX660. [file FEB4-11-714-s001.pdf]

Supplementary Figure 1: Absorbance spectrum of ASTX660

Absorbance spectrum of ASTX660 was determined to rule out potential interference with MTT-based viability assays, which are measured at 595 nm (wavelength depicted as vertical blue line in the pictures below). Absorbance of ASTX660 at 595 nm in DMSO (Supplementary Figure 1A) and medium (Supplementary Figure 1B) was negligible. Thus, interference of ASTX660 with MTT viability assays due to the absorbance spectrum of ASTX660 is unlikely.

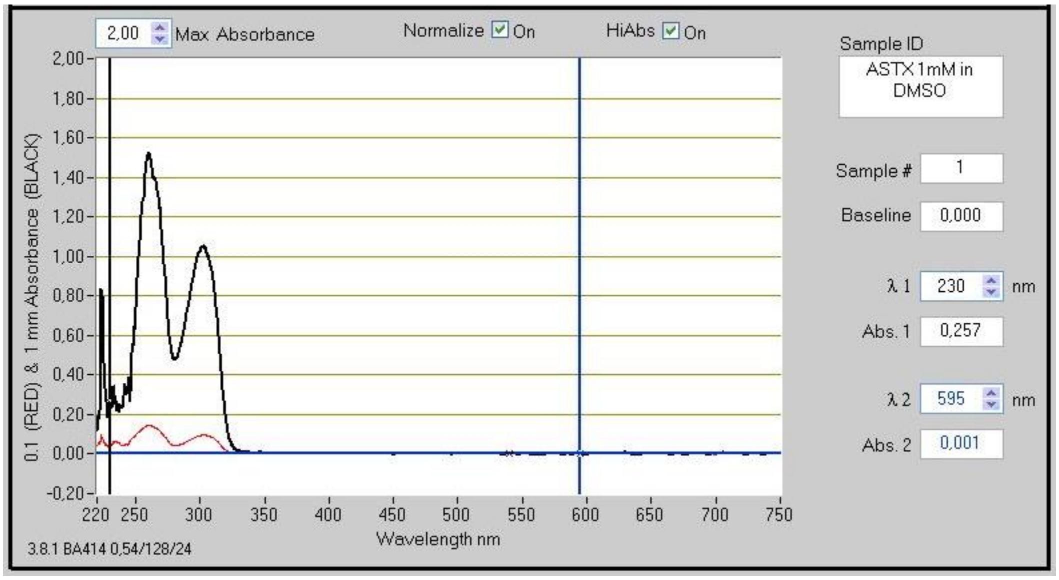

Supplementary Figure 1A: Absorbance spectrum of ASTX660 (1 mM) in DMSO was measured using a NanoDrop 1000 Spectrophotometer (Thermo Fisher Scientific, Wilmington, DE, USA).

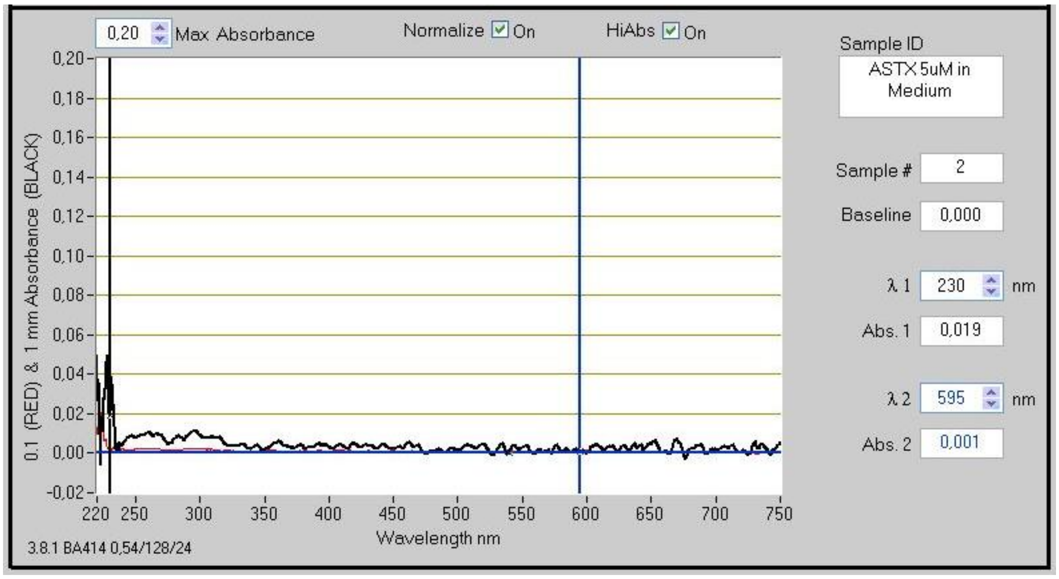

Supplementary Figure 1B: Absorbance spectrum of ASTX660 (5 µM) in medium.
