# Supplementary material for: The non‐peptidomimetic IAP antagonist ASTX660 sensitizes colorectal cancer cells for extrinsic apoptosis
Source: FEBS Open Bio. 2021 Feb 19;11(3):714–23. doi: 10.1002/2211-5463.13096 (PMC7931242; doi:10.1002/2211-5463.13096)
Supplement: Supplementary file 2 — Table S1. Numeric values ± SD to Fig. 1A. Table S2. Numeric values ± SD to Fig. 2A. Table S3. Numeric values ± SD to Fig. 2B. Table S4. Numeric values ± SD to Fig. 4E. Table S5. Numeric values ± SD to Fig. 5A‐D. Table S6. Numeric values ± SD to Fig. 3A. Table S7. Numeric values ± SD to Fig. 3B. [file FEB4-11-714-s002.pdf]

Supplementary Table 1 (to Figure 1A)

	ASTX660	n	mean (%)	SD (%)
**Colo205**	0 µM	3	100.00	--
	0.075 µM	3	99.18	3.16
	0.15 µM	3	97.36	3.74
	0.3 µM	3	96.93	3.51
	0.6 µM	3	95.72	3.39
	1.25 µM	3	94.32	3.38
	2.5 µM	3	97.38	5.54
	5 µM	3	96.03	8.94
	10 µM	3	102.99	9.72
**DLD1**	0 µM	3	100.00	---
	0.075 µM	3	100.45	0.40
	0.15 µM	3	100.13	1.15
	0.3 µM	3	100.22	0.79
	0.6 µM	3	101.71	1.20
	1.25 µM	3	103.32	0.16
	2.5 µM	3	101.35	0.75
	5 µM	3	101.88	1.22
	10 µM	3	104.14	0.88
**HCT8**	0 µM	3	100.00	---
	0.075 µM	3	99.41	2.56
	0.15 µM	3	99.94	3.05
	0.3 µM	3	99.56	2.39
	0.6 µM	3	99.47	3.97
	1.25 µM	3	101.82	2.70
	2.5 µM	3	100.86	1.50
	5 µM	3	103.05	2.20
	10 µM	3	104.47	5.49
**HCT116**	0 µM	3	100.00	---
	0.075 µM	3	97.29	0.52
	0.15 µM	3	96.43	1.33
	0.3 µM	3	99.88	7.54
	0.6 µM	3	98.46	0.88
	1.25 µM	3	97.38	0.58
	2.5 µM	3	100.39	5.18
	5 µM	3	96.99	2.11
	10 µM	3	99.60	1.37
**HT29**	0 µM	3	100.00	---
	0.075 µM	3	104.20	21.37
	0.15 µM	3	100.24	9.76
	0.3 µM	3	97.92	11.00
	0.6 µM	3	94.66	8.63
	1.25 µM	3	97.20	12.84
	2.5 µM	3	93.32	6.56
	5 µM	3	97.51	12.66
	10 µM	3	104.94	13.24
**LoVo**	0 µM	3	100.00	---
	0.075 µM	3	96.07	2.85
	0.15 µM	3	93.49	4.64
	0.3 µM	3	97.07	2.88
	0.6 µM	3	93.03	8.03
	1.25 µM	3	95.55	2.97
	2.5 µM	3	92.54	7.33
	5 µM	3	95.22	4.99
	10 µM	3	97.70	4.51

	ASTX660	n	mean (%)	SD (%)
**LS174T**	0 µM	3	100.00	---
	0.075 µM	3	104.95	4.63
	0.15 µM	3	101.81	4.48
	0.3 µM	3	101.64	2.58
	0.6 µM	3	102.17	3.30
	1.25 µM	3	103.18	4.64
	2.5 µM	3	103.78	3.07
	5 µM	3	104.38	3.60
	10 µM	3	104.15	6.81
**RKO**	0 µM	3	100.00	---
	0.075 µM	3	98.44	1.79
	0.15 µM	3	96.77	0.60
	0.3 µM	3	95.52	0.75
	0.6 µM	3	95.32	2.50
	1.25 µM	3	96.56	1.73
	2.5 µM	3	96.75	2.43
	5 µM	3	99.85	3.04
	10 µM	3	102.51	7.43
**SW48**	0 µM	3	100.00	---
	0.075 µM	3	98.29	0.65
	0.15 µM	3	98.04	0.44
	0.3 µM	3	97.46	0.49
	0.6 µM	3	97.19	1.43
	1.25 µM	3	98.26	1.92
	2.5 µM	3	99.49	0.28
	5 µM	3	100.24	1.12
	10 µM	3	105.01	1.12
**SW480**	0 µM	3	100.00	---
	0.075 µM	3	100.09	2.05
	0.15 µM	3	99.39	2.07
	0.3 µM	3	99.98	2.18
	0.6 µM	3	100.53	2.50
	1.25 µM	3	101.58	3.37
	2.5 µM	3	102.31	2.38
	5 µM	3	106.13	2.16
	10 µM	3	111.07	2.06
**SW948**	0 µM	3	100.00	---
	0.075 µM	3	108.65	7.04
	0.15 µM	3	108.61	2.33
	0.3 µM	3	115.05	8.37
	0.6 µM	3	116.32	9.49
	1.25 µM	3	120.49	7.89
	2.5 µM	3	126.29	7.80
	5 µM	3	134.51	11.01
	10 µM	3	125.40	3.15

Supplementary Table 2 (to Figure 2A)

	KillerTRAIL	control n	control mean (%)	control SD (%)	1.25 µM ASTX660 n	1.25 µM ASTX660 mean (%)	1.25 µM ASTX660 SD (%)	1.25 µM Birinapant n	1.25 µM Birinapant mean (%)	1.25 µM Birinapant SD (%)	5 µM ASTX660 n	5 µM ASTX660 mean (%)	5 µM ASTX660 SD (%)	5 µM Birinapant n	5 µM Birinapant mean (%)	5 µM Birinapant SD (%)
**Colo205**	0 ng/mL	3	100.00	---	3	102.23	6.39	3	98.26	4.26	3	125.65	8.93	3	112.55	2.02
	2 ng/mL	3	106.63	3.14	3	65.83	17.35	3	37.70	12.62	3	80.43	13.94	3	49.28	12.27
	4 ng/mL	3	98.34	8.39	3	47.08	15.82	3	27.19	10.42	3	57.29	14.09	3	37.66	9.83
	8 ng/mL	3	85.74	14.62	3	30.72	13.35	3	20.93	9.62	3	42.95	11.43	3	32.94	8.59
	16 ng/mL	3	63.72	13.76	3	22.29	11.53	3	18.31	8.83	3	34.68	10.01	3	31.52	6.93
	32 ng/mL	3	33.46	14.29	3	17.74	10.21	3	15.56	9.14	3	31.25	8.75	3	28.81	7.74
	64 ng/mL	3	22.62	9.30	3	15.69	9.93	3	14.23	8.73	3	30.20	10.44	3	27.69	7.22
	128 ng/mL	3	17.11	9.57	3	14.13	9.53	3	13.01	8.76	3	27.56	9.76	3	26.08	9.09
	256 ng/mL	3	16.47	9.64	3	13.90	9.94	3	11.96	8.00	3	26.17	9.15	3	24.24	8.79
**DLD1**	0 ng/mL	3	100.00	---	3	97.12	6.49	3	98.10	11.37	3	100.67	10.02	3	103.29	5.10
	2 ng/mL	3	79.79	10.42	3	59.12	6.60	3	63.56	10.82	3	63.47	9.06	3	57.35	10.23
	4 ng/mL	3	72.17	7.05	3	49.80	1.74	3	52.27	8.90	3	51.84	8.76	3	45.31	10.71
	8 ng/mL	3	57.91	4.07	3	37.06	2.54	3	37.32	8.67	3	39.87	7.31	3	34.68	10.36
	16 ng/mL	3	41.11	5.42	3	25.02	4.92	3	24.71	8.42	3	26.56	7.99	3	22.41	9.08
	32 ng/mL	3	28.13	5.20	3	18.11	4.28	3	17.67	6.68	3	20.32	6.88	3	17.60	7.42
	64 ng/mL	3	20.23	3.78	3	14.73	4.33	3	14.75	5.81	3	17.42	6.96	3	15.29	6.12
	128 ng/mL	3	15.62	4.79	3	12.53	4.21	3	13.40	5.97	3	15.43	7.45	3	14.35	6.47
	256 ng/mL	3	16.05	4.90	3	13.46	4.60	3	14.04	6.00	3	15.60	6.48	3	15.16	6.17
**HCT8**	0 ng/mL	3	100.00	---	3	93.75	1.35	3	92.61	5.32	3	98.24	2.37	3	97.86	7.48
	2 ng/mL	3	90.25	3.39	3	80.49	2.74	3	79.72	4.25	3	83.24	3.77	3	74.52	6.23
	4 ng/mL	3	87.37	1.40	3	72.04	5.89	3	70.73	5.49	3	75.36	4.04	3	65.64	4.15
	8 ng/mL	3	83.13	4.67	3	62.42	2.59	3	57.66	3.64	3	66.51	4.27	3	55.08	8.56
	16 ng/mL	3	71.00	6.27	3	49.05	5.21	3	44.52	6.29	3	52.35	5.16	3	39.92	10.44
	32 ng/mL	3	56.12	5.90	3	37.60	5.46	3	32.11	6.04	3	41.37	5.48	3	29.85	8.71
	64 ng/mL	3	44.08	4.04	3	29.86	4.01	3	24.54	4.84	3	33.33	4.74	3	23.05	6.89
	128 ng/mL	3	31.49	4.73	3	23.88	4.45	3	19.29	4.45	3	26.38	5.88	3	18.73	5.56
	256 ng/mL	3	31.17	3.33	3	24.06	4.01	3	19.85	4.34	3	26.16	3.77	3	19.64	5.75
**HCT116**	0 ng/mL	3	100.00	---	3	92.44	8.90	3	91.78	2.48	3	101.38	6.16	3	92.59	5.92
	2 ng/mL	3	81.52	8.92	3	61.04	11.87	3	52.27	15.35	3	62.96	12.69	3	48.18	14.80
	4 ng/mL	3	74.97	9.97	3	51.64	10.88	3	39.09	12.69	3	52.75	12.59	3	36.61	12.01
	8 ng/mL	3	61.61	13.00	3	41.10	10.42	3	28.60	10.21	3	44.53	10.66	3	26.85	10.63
	16 ng/mL	3	45.35	13.78	3	33.41	9.28	3	20.76	7.33	3	34.78	9.85	3	20.33	8.40
	32 ng/mL	3	34.44	9.84	3	27.89	8.22	3	17.18	6.55	3	30.22	7.81	3	16.97	6.49
	64 ng/mL	3	28.67	7.86	3	24.46	7.31	3	15.84	6.42	3	25.98	7.03	3	15.76	5.98
	128 ng/mL	3	25.25	7.25	3	22.12	6.62	3	14.47	5.81	3	23.24	6.72	3	14.56	5.76
	256 ng/mL	3	25.38	6.92	3	21.87	6.24	3	14.38	5.33	3	23.19	6.61	3	14.71	5.63

Supplementary Table 2 (continued, to Figure 2A)

		control			1.25 µM ASTX660			1.25 µM Birinapant			5 µM ASTX660			5 µM Birinapant		
	KillerTRAIL	n	mean (%)	SD (%)	n	mean (%)	SD (%)	n	mean (%)	SD (%)	n	mean (%)	SD (%)	n	mean (%)	SD (%)
**HT29**	0 ng/mL	3	100.00	---	3	98.89	5.28	3	91.58	11.21	3	112.49	6.12	3	104.13	8.07
	2 ng/mL	3	100.05	7.02	3	88.92	6.77	3	50.24	5.96	3	97.82	7.38	3	49.06	3.87
	4 ng/mL	3	96.92	4.26	3	83.12	4.32	3	43.19	4.07	3	91.72	5.53	3	39.07	2.64
	8 ng/mL	3	93.65	5.24	3	75.78	3.00	3	36.64	2.77	3	83.39	1.46	3	33.30	2.39
	16 ng/mL	3	93.64	2.88	3	69.31	1.46	3	31.33	3.00	3	78.38	3.34	3	29.17	1.66
	32 ng/mL	3	87.91	2.88	3	60.41	3.18	3	24.99	1.61	3	67.77	1.36	3	24.27	1.69
	64 ng/mL	3	81.86	4.94	3	56.74	2.48	3	21.19	0.87	3	61.42	2.86	3	21.41	1.07
	128 ng/mL	3	83.01	2.51	3	53.76	2.58	3	18.53	0.18	3	59.17	1.01	3	19.11	0.72
	256 ng/mL	3	82.67	3.44	3	52.36	0.99	3	18.13	0.19	3	57.71	0.47	3	18.73	0.67
**LoVo**	0 ng/mL	3	100.00	---	3	96.82	2.63	3	95.01	3.24	3	103.72	5.82	3	101.81	4.19
	2 ng/mL	3	96.00	0.90	3	80.26	4.30	3	81.54	4.73	3	87.22	2.97	3	84.26	7.54
	4 ng/mL	3	93.78	3.18	3	78.28	5.97	3	76.93	6.91	3	83.91	4.29	3	77.19	5.89
	8 ng/mL	3	92.79	1.54	3	74.00	2.92	3	71.87	7.66	3	80.06	2.63	3	70.87	5.87
	16 ng/mL	3	87.43	1.69	3	72.25	2.96	3	63.36	3.07	3	77.98	2.32	3	60.24	5.53
	32 ng/mL	3	81.34	0.69	3	65.99	3.63	3	48.81	4.27	3	72.00	3.86	3	45.39	4.46
	64 ng/mL	3	73.96	5.26	3	56.92	5.38	3	37.20	2.60	3	66.45	6.39	3	34.44	3.79
	128 ng/mL	3	65.45	4.64	3	50.74	4.51	3	28.34	1.10	3	58.02	4.04	3	23.15	10.30
	256 ng/mL	3	61.49	5.10	3	45.12	5.54	3	23.38	0.82	3	52.36	5.79	3	23.86	4.16
**LS174T**	0 ng/mL	3	100.00	--	3	96.20	4.29	3	94.56	4.33	3	104.46	8.79	3	100.09	1.84
	2 ng/mL	3	102.77	7.70	3	88.46	5.58	3	61.77	6.46	3	90.86	7.08	3	58.02	5.45
	4 ng/mL	3	95.76	5.85	3	78.62	5.75	3	45.92	7.65	3	81.54	7.86	3	40.07	3.96
	8 ng/mL	3	93.58	4.99	3	60.81	3.17	3	24.69	3.77	3	66.55	6.86	3	24.91	1.83
	16 ng/mL	3	77.85	6.78	3	38.08	0.96	3	13.40	0.80	3	43.02	1.25	3	15.83	1.28
	32 ng/mL	3	51.54	1.52	3	20.47	1.02	3	9.07	0.83	3	25.56	1.37	3	12.12	1.15
	64 ng/mL	3	25.37	4.71	3	12.60	0.80	3	7.49	0.74	3	16.70	1.57	3	10.77	1.06
	128 ng/mL	3	15.85	1.03	3	8.92	1.05	3	6.41	1.15	3	12.88	0.69	3	9.36	0.77
	256 ng/mL	3	12.07	0.32	3	8.12	1.33	3	6.22	1.30	3	11.51	0.83	3	8.87	0.76
**RKO**	0 ng/mL	3	100.00	---	3	94.29	2.51	3	89.70	1.94	3	110.12	6.30	3	101.51	1.56
	2 ng/mL	3	96.54	1.69	3	86.47	0.72	3	66.15	7.59	3	99.97	6.04	3	76.41	7.90
	4 ng/mL	3	93.94	0.59	3	83.89	2.99	3	62.08	11.02	3	96.99	5.11	3	72.94	8.85
	8 ng/mL	3	94.14	0.88	3	83.95	0.66	3	59.53	10.33	3	96.30	4.56	3	71.02	9.71
	16 ng/mL	3	93.04	1.11	3	82.99	0.84	3	57.64	11.53	3	97.29	5.67	3	70.91	8.67
	32 ng/mL	3	91.85	1.18	3	79.57	2.22	3	53.81	13.08	3	94.96	5.82	3	67.97	10.21
	64 ng/mL	3	87.39	2.55	3	74.41	3.92	3	51.31	12.32	3	88.34	2.44	3	65.59	12.69
	128 ng/mL	3	86.68	3.23	3	71.80	4.86	3	49.35	14.20	3	84.69	3.19	3	61.73	14.61
	256 ng/mL	3	89.96	2.24	3	72.87	4.62	3	50.39	12.71	3	84.47	3.26	3	62.42	17.18

Supplementary Table 2 (continued, to Figure 2A)

		control			1.25 µM ASTX660			1.25 µM Birinapant			5 µM ASTX660			5 µM Birinapant		
	KillerTRAIL	n	mean (%)	SD (%)	n	mean (%)	SD (%)	n	mean (%)	SD (%)	n	mean (%)	SD (%)	n	mean (%)	SD (%)
**SW48**	0 ng/mL	3	100.00	---	3	99.52	4.20	3	98.17	2.56	3	98.88	6.67	3	97.52	6.58
	2 ng/mL	3	96.43	1.68	3	66.09	10.91	3	44.22	14.44	3	64.63	13.78	3	45.54	18.78
	4 ng/mL	3	89.93	4.62	3	51.76	10.06	3	32.13	10.48	3	51.09	12.14	3	32.18	13.53
	8 ng/mL	3	73.58	8.97	3	43.76	8.53	3	25.76	7.47	3	43.51	9.35	3	26.89	11.36
	16 ng/mL	3	56.54	8.75	3	38.81	7.88	3	21.96	6.38	3	37.00	8.77	3	23.11	11.42
	32 ng/mL	3	48.68	9.22	3	34.65	7.80	3	20.08	7.25	3	34.29	8.85	3	21.51	11.65
	64 ng/mL	3	43.80	8.27	3	32.49	10.09	3	17.69	5.83	3	31.23	8.51	3	19.31	10.95
	128 ng/mL	3	40.18	7.53	3	28.79	8.02	3	15.94	6.41	3	28.31	8.46	3	16.94	9.65
	256 ng/mL	3	39.08	7.62	3	28.42	7.87	3	15.46	4.94	3	26.76	7.41	3	16.79	9.16
**SW480**	0 ng/mL	3	100.00	---	3	100.45	4.07	3	95.58	3.90	3	109.38	6.76	3	106.29	4.44
	2 ng/mL	3	96.51	2.55	3	85.19	1.76	3	76.20	2.06	3	96.88	4.50	3	85.60	5.28
	4 ng/mL	3	93.75	0.32	3	81.31	3.27	3	67.95	1.87	3	91.32	6.86	3	73.45	7.69
	8 ng/mL	3	91.07	1.21	3	76.40	3.45	3	54.28	5.19	3	83.51	6.36	3	59.10	8.71
	16 ng/mL	3	84.90	0.08	3	66.68	4.09	3	39.80	5.66	3	77.85	5.30	3	42.84	7.17
	32 ng/mL	3	76.74	1.85	3	60.11	4.22	3	25.38	6.05	3	67.90	6.97	3	29.97	5.51
	64 ng/mL	3	65.97	3.35	3	51.53	4.28	3	18.17	5.14	3	58.88	7.66	3	23.45	5.51
	128 ng/mL	3	56.90	3.53	3	43.71	5.28	3	14.31	3.81	3	51.48	8.35	3	19.56	3.94
	256 ng/mL	3	50.58	4.84	3	38.70	4.70	3	13.23	3.85	3	45.12	7.53	3	17.65	3.09
**SW948**	0 ng/mL	3	100.00	---	3	104.21	6.19	3	90.12	7.16	3	112.38	4.56	3	95.30	14.24
	2 ng/mL	3	98.82	10.53	3	81.34	17.44	3	74.18	15.23	3	83.38	14.63	3	64.08	10.89
	4 ng/mL	3	81.13	15.78	3	63.73	14.88	3	62.81	15.35	3	72.49	20.69	3	61.46	16.77
	8 ng/mL	3	84.31	27.34	3	58.87	24.46	3	53.47	21.30	3	61.45	24.83	3	51.18	14.92
	16 ng/mL	3	61.86	33.31	3	42.40	20.62	3	43.18	15.24	3	50.13	26.43	3	44.72	12.40
	32 ng/mL	3	40.38	20.09	3	25.25	8.45	3	27.91	10.98	3	31.15	6.65	3	32.48	4.12
	64 ng/mL	3	23.82	10.49	3	22.13	7.53	3	20.29	4.95	3	28.91	9.32	3	26.94	2.23
	128 ng/mL	3	22.51	6.30	3	20.03	5.66	3	19.72	4.56	3	25.76	6.41	3	25.14	0.47
	256 ng/mL	3	19.64	3.48	3	17.86	3.92	3	17.44	2.02	3	19.40	4.02	3	20.70	3.46

Supplementary Table 3 (to Figure 2B)

		control			1.25 µM ASTX660			1.25 µM Birinapant			5 µM ASTX660			5 µM Birinapant		
	TNF	n	mean (%)	SD (%)	n	mean (%)	SD (%)	n	mean (%)	SD (%)	n	mean (%)	SD (%)	n	mean (%)	SD (%)
Colo205	0 ng/mL	3	100.00	--	3	99.16	7.27	3	97.39	9.80	3	116.44	10.35	3	116.25	8.84
	2 ng/mL	3	104.40	9.31	3	30.55	11.44	3	16.49	9.37	3	40.19	9.69	3	28.64	9.00
	4 ng/mL	3	98.41	16.75	3	29.46	11.91	3	16.28	9.52	3	38.37	10.22	3	28.57	8.13
	8 ng/mL	3	103.19	8.32	3	28.94	11.80	3	15.95	9.41	3	38.21	9.79	3	28.65	7.38
	16 ng/mL	3	100.81	8.45	3	28.51	11.49	3	16.49	9.77	3	38.61	10.19	3	30.83	7.05
	32 ng/mL	3	102.53	10.02	3	27.92	11.84	3	15.76	9.70	3	38.32	10.00	3	30.29	7.74
	64 ng/mL	3	100.62	11.90	3	27.55	11.90	3	15.81	9.66	3	39.14	10.96	3	30.90	8.68
	128 ng/mL	3	100.29	10.92	3	26.96	11.22	3	15.36	9.10	3	37.26	10.23	3	28.58	9.02
	256 ng/mL	3	98.82	13.07	3	28.49	10.90	3	15.50	9.97	3	37.56	9.43	3	27.06	8.14
DLD1	0 ng/mL	3	100.00	---	3	99.45	6.03	3	95.65	7.50	3	99.88	2.94	3	99.93	2.73
	2 ng/mL	3	95.77	4.46	3	75.81	4.03	3	64.78	2.72	3	72.38	4.41	3	55.92	3.30
	4 ng/mL	3	94.50	2.41	3	77.72	6.71	3	60.34	6.23	3	73.05	0.78	3	54.97	1.15
	8 ng/mL	3	94.35	1.49	3	73.84	2.46	3	60.25	7.40	3	76.22	4.00	3	53.60	1.13
	16 ng/mL	3	94.08	1.37	3	72.76	4.14	3	63.50	6.23	3	70.69	3.16	3	53.32	1.57
	32 ng/mL	3	93.25	6.52	3	72.62	3.88	3	61.94	6.58	3	74.02	5.19	3	54.22	2.97
	64 ng/mL	3	92.40	4.22	3	74.20	8.96	3	62.75	8.63	3	73.34	5.87	3	54.18	4.31
	128 ng/mL	3	91.81	3.06	3	75.53	4.33	3	62.57	10.35	3	70.92	8.19	3	52.56	2.41
	256 ng/mL	3	94.61	4.88	3	74.49	6.44	3	63.03	9.62	3	76.84	3.52	3	55.12	5.42
HCT8	0 ng/mL	3	100.00	---	3	91.01	4.24	3	95.23	2.42	3	93.40	7.95	3	91.49	6.90
	2 ng/mL	3	89.79	6.65	3	78.96	5.08	3	75.83	3.83	3	74.68	2.48	3	62.86	6.98
	4 ng/mL	3	89.71	9.11	3	73.59	3.13	3	74.40	1.60	3	75.94	4.30	3	65.77	3.37
	8 ng/mL	3	87.56	4.44	3	73.02	2.18	3	72.95	2.29	3	78.17	1.59	3	65.03	5.26
	16 ng/mL	3	82.68	3.64	3	78.38	4.09	3	73.05	5.40	3	75.30	3.42	3	65.31	5.74
	32 ng/mL	3	88.64	4.12	3	77.40	2.65	3	73.34	4.85	3	77.55	2.76	3	66.62	6.88
	64 ng/mL	3	89.73	5.17	3	77.20	2.19	3	73.22	3.58	3	77.15	2.24	3	65.35	8.30
	128 ng/mL	3	85.16	4.11	3	79.12	6.77	3	74.57	5.24	3	76.62	1.86	3	64.63	7.66
	256 ng/mL	3	93.77	2.11	3	79.96	1.68	3	78.71	9.32	3	79.58	0.40	3	67.26	10.88
HCT116	0 ng/mL	3	100.00	---	3	96.99	3.49	3	96.02	4.84	3	97.47	3.39	3	94.76	5.23
	2 ng/mL	3	90.17	3.80	3	69.46	1.05	3	53.44	4.51	3	69.31	5.54	3	47.39	4.01
	4 ng/mL	3	90.51	5.34	3	66.34	3.78	3	54.80	3.88	3	68.15	5.06	3	48.01	2.83
	8 ng/mL	3	88.50	4.08	3	66.89	1.86	3	53.19	4.96	3	69.96	2.63	3	50.26	3.53
	16 ng/mL	3	87.60	4.28	3	66.69	2.94	3	53.32	4.16	3	66.74	3.74	3	48.22	4.38
	32 ng/mL	3	85.59	3.59	3	64.09	4.53	3	53.26	3.55	3	66.62	3.80	3	49.25	5.12
	64 ng/mL	3	86.01	5.36	3	64.48	5.11	3	53.94	4.09	3	65.87	5.34	3	49.58	4.33
	128 ng/mL	3	88.16	2.70	3	66.89	4.12	3	54.82	3.02	3	63.71	5.69	3	47.95	5.29
	256 ng/mL	3	87.52	3.34	3	63.71	5.70	3	52.31	5.79	3	64.48	5.66	3	47.69	5.65

Supplementary Table 3 (continued, to Figure 2B)

		control			1.25 µM ASTX660			1.25 µM Birinapant			5 µM ASTX660			5 µM Birinapant		
	TNF	n	mean (%)	SD (%)	n	mean (%)	SD (%)	n	mean (%)	SD (%)	n	mean (%)	SD (%)	n	mean (%)	SD (%)
**HT29**	0 ng/mL	3	100.00	---	3	96.96	6.55	3	91.15	9.26	3	107.04	8.22	3	99.66	10.79
	2 ng/mL	3	101.55	7.45	3	86.75	12.22	3	55.15	6.41	3	96.95	9.28	3	53.92	5.23
	4 ng/mL	3	95.59	10.89	3	86.37	9.22	3	55.34	6.19	3	90.07	8.58	3	54.71	4.34
	8 ng/mL	3	93.16	11.70	3	84.29	10.91	3	57.28	3.68	3	91.57	9.34	3	55.93	4.21
	16 ng/mL	3	89.73	8.37	3	85.78	9.92	3	56.71	4.96	3	89.43	11.51	3	57.64	5.20
	32 ng/mL	3	93.46	11.45	3	83.34	11.44	3	55.78	5.29	3	86.27	10.49	3	54.58	4.11
	64 ng/mL	3	91.71	10.28	3	81.93	12.41	3	54.71	8.24	3	85.97	8.74	3	54.38	4.33
	128 ng/mL	3	92.23	7.70	3	81.62	7.86	3	56.24	7.27	3	86.51	9.88	3	55.03	5.50
	256 ng/mL	3	94.89	5.66	3	83.56	5.90	3	54.41	5.12	3	87.41	7.20	3	56.86	4.46
**LoVo**	0 ng/mL	3	100.00	---	3	95.99	3.59	3	95.72	1.51	3	103.10	6.39	3	105.28	3.73
	2 ng/mL	3	96.99	5.03	3	85.12	6.29	3	84.57	4.47	3	92.84	4.22	3	91.12	1.68
	4 ng/mL	3	93.41	5.25	3	84.20	5.08	3	85.20	4.17	3	90.55	4.39	3	88.32	1.10
	8 ng/mL	3	91.58	5.75	3	84.42	3.52	3	82.55	8.56	3	90.50	3.94	3	88.57	2.66
	16 ng/mL	3	90.38	6.25	3	82.10	5.54	3	81.79	8.48	3	88.54	4.59	3	87.29	5.14
	32 ng/mL	3	89.16	7.01	3	82.33	7.01	3	83.25	4.73	3	89.94	2.21	3	87.62	2.47
	64 ng/mL	3	87.96	7.55	3	82.45	6.73	3	85.53	3.15	3	90.22	4.22	3	86.12	4.73
	128 ng/mL	3	88.60	8.25	3	83.03	6.95	3	87.06	4.18	3	89.25	3.64	3	85.77	3.25
	256 ng/mL	3	91.48	6.80	3	83.76	5.23	3	84.51	6.06	3	91.81	3.66	3	87.31	6.04
**LS174T**	0 ng/mL	3	100.00	---	3	97.91	2.21	3	94.45	9.48	3	107.58	7.36	3	101.74	5.00
	2 ng/mL	3	100.59	10.28	3	86.85	7.27	3	42.97	8.11	3	90.93	2.79	3	38.31	10.01
	4 ng/mL	3	97.60	7.78	3	87.31	6.76	3	43.47	7.42	3	87.72	5.37	3	38.57	10.08
	8 ng/mL	3	101.26	12.55	3	87.98	7.43	3	43.48	6.78	3	92.34	6.57	3	39.22	10.90
	16 ng/mL	3	100.08	11.40	3	88.06	6.21	3	42.87	7.51	3	92.71	5.82	3	39.38	11.73
	32 ng/mL	3	100.04	9.55	3	88.84	4.76	3	43.49	7.88	3	91.02	2.72	3	39.40	11.54
	64 ng/mL	3	100.50	8.37	3	86.74	7.31	3	43.44	8.33	3	92.65	7.40	3	38.88	10.34
	128 ng/mL	3	102.13	10.01	3	88.87	5.92	3	42.55	9.32	3	91.36	4.42	3	40.08	11.76
	256 ng/mL	3	99.55	8.69	3	84.79	5.42	3	44.23	10.61	3	88.71	4.61	3	40.39	12.89
**RKO**	0 ng/mL	3	100.00	---	3	96.30	3.02	3	89.86	2.38	3	111.44	4.78	3	101.78	1.79
	2 ng/mL	3	96.02	1.08	3	88.54	1.46	3	70.93	5.59	3	101.38	4.01	3	79.71	4.75
	4 ng/mL	3	93.89	2.51	3	87.17	1.54	3	69.76	5.76	3	102.19	4.51	3	79.73	5.86
	8 ng/mL	3	91.77	3.79	3	86.01	4.58	3	69.62	7.48	3	100.60	6.17	3	80.47	5.60
	16 ng/mL	3	92.80	2.53	3	85.92	5.05	3	70.86	6.38	3	100.75	4.76	3	80.74	7.72
	32 ng/mL	3	91.46	3.15	3	85.83	5.76	3	71.05	7.43	3	99.40	7.13	3	82.37	7.27
	64 ng/mL	3	93.24	2.56	3	87.35	5.65	3	72.46	8.19	3	100.32	3.29	3	82.63	7.27
	128 ng/mL	3	90.75	6.24	3	86.99	9.19	3	72.55	8.79	3	99.42	7.19	3	81.95	7.91
	256 ng/mL	3	97.30	6.31	3	90.47	7.42	3	76.29	8.62	3	103.23	6.64	3	84.21	10.79

Supplementary Table 3 (continued, to Figure 2B)

		control			1.25 µM ASTX660			1.25 µM Birinapant			5 µM ASTX660			5 µM Birinapant		
	TNF	n	mean (%)	SD (%)	n	mean (%)	SD (%)	n	mean (%)	SD (%)	n	mean (%)	SD (%)	n	mean (%)	SD (%)
**SW48**	0 ng/mL	3	100.00	---	3	98.00	2.08	3	96.69	3.79	3	103.25	5.63	3	100.31	7.54
	2 ng/mL	3	104.17	0.37	3	68.93	3.38	3	34.46	8.32	3	66.56	3.86	3	30.86	6.47
	4 ng/mL	3	104.29	1.00	3	69.84	3.89	3	35.83	8.44	3	65.70	5.40	3	31.88	6.41
	8 ng/mL	3	102.48	2.52	3	67.34	4.76	3	34.93	7.75	3	66.78	5.34	3	32.92	6.56
	16 ng/mL	3	102.14	1.78	3	66.33	8.19	3	35.99	8.34	3	64.59	5.10	3	32.28	6.89
	32 ng/mL	3	103.40	0.88	3	66.68	6.41	3	35.98	8.21	3	65.62	5.17	3	32.92	6.98
	64 ng/mL	3	102.68	0.61	3	67.48	5.81	3	37.01	8.50	3	66.25	6.12	3	33.00	6.73
	128 ng/mL	3	103.19	2.05	3	67.00	7.13	3	37.57	8.73	3	64.78	5.52	3	32.31	6.62
	256 ng/mL	3	107.93	2.95	3	68.37	5.71	3	36.02	8.24	3	61.03	11.17	3	31.85	6.85
**SW480**	0 ng/mL	3	100.00	---	3	100.79	0.33	3	96.59	2.49	3	108.21	5.48	3	108.03	3.14
	2 ng/mL	3	100.59	2.76	3	89.62	5.51	3	76.07	3.59	3	94.48	4.14	3	81.86	6.02
	4 ng/mL	3	97.22	3.41	3	88.34	3.12	3	74.61	3.15	3	92.20	5.86	3	79.20	5.84
	8 ng/mL	3	97.73	2.19	3	88.33	1.60	3	74.48	2.37	3	91.24	5.85	3	78.70	6.25
	16 ng/mL	3	98.49	2.55	3	87.98	0.94	3	73.92	0.79	3	92.36	5.25	3	78.52	4.34
	32 ng/mL	3	98.36	1.77	3	88.23	1.35	3	73.76	2.27	3	91.53	4.43	3	79.01	5.68
	64 ng/mL	3	96.15	4.88	3	87.18	1.67	3	74.19	0.92	3	91.83	5.02	3	78.07	6.55
	128 ng/mL	3	97.80	4.74	3	85.98	0.89	3	75.04	1.48	3	93.17	2.86	3	78.41	6.03
	256 ng/mL	3	100.55	4.40	3	91.40	5.24	3	76.29	4.61	3	95.50	1.73	3	79.61	5.98
**SW948**	0 ng/mL	3	100.00	---	3	95.76	2.67	3	89.89	7.56	3	108.18	1.48	3	105.34	9.73
	2 ng/mL	3	102.07	2.95	3	82.95	4.86	3	90.13	15.41	3	85.15	3.43	3	83.64	8.70
	4 ng/mL	3	92.96	3.19	3	85.01	6.30	3	87.61	14.25	3	92.58	4.25	3	85.89	9.17
	8 ng/mL	3	102.36	5.95	3	88.61	1.99	3	101.21	22.45	3	92.80	8.47	3	87.86	13.27
	16 ng/mL	3	104.53	4.19	3	89.01	5.71	3	99.87	20.26	3	98.92	7.00	3	93.89	15.24
	32 ng/mL	3	107.03	5.54	3	91.12	8.35	3	100.98	20.25	3	94.14	6.50	3	97.89	16.63
	64 ng/mL	3	108.88	3.92	3	95.77	6.77	3	106.93	23.17	3	103.73	13.18	3	102.21	17.36
	128 ng/mL	3	121.32	12.77	3	105.45	14.04	3	113.38	31.29	3	104.33	13.89	3	98.88	15.66
	256 ng/mL	3	123.82	5.79	3	91.66	1.08	3	97.23	14.72	3	84.92	9.41	3	79.31	17.79

Supplementary Table 4 (to Figure 4E)

		2.5 µM ASTX660			2.5 µM ASTX660 + 50 µM QVD-OPh			2.5 µM Birinapant			2.5 µM Birinapant + 50 µM QVD-OPh		
	KillerTRAIL	n	mean (%)	SD (%)	n	mean (%)	SD (%)	n	mean (%)	SD (%)	n	mean (%)	SD (%)
DLD1	0 ng/mL	3	100.00	---	3	104.06	3.42	3	100.00	---	3	104.10	3.59
	2 ng/mL	3	91.18	1.26	3	99.84	5.24	3	86.21	5.40	3	99.25	7.79
	4 ng/mL	3	82.85	5.69	3	96.30	7.38	3	76.69	3.74	3	102.85	10.94
	8 ng/mL	3	69.05	4.43	3	98.14	8.18	3	67.33	2.64	3	98.01	9.54
	16 ng/mL	3	56.81	3.18	3	100.01	11.63	3	48.91	5.86	3	102.47	10.05
	32 ng/mL	3	39.54	2.23	3	92.86	7.23	3	30.40	1.58	3	101.70	7.59
	64 ng/mL	3	28.93	1.21	3	94.64	5.16	3	22.93	1.33	3	101.45	8.26
	128 ng/mL	3	23.44	1.78	3	97.81	3.05	3	18.69	1.46	3	98.98	7.24
	256 ng/mL	3	20.93	1.46	3	100.39	3.45	3	18.30	2.10	3	98.93	2.58
HCT116	0 ng/mL	3	100.00	---	3	96.24	1.28	3	100.00	---	3	102.81	9.65
	2 ng/mL	3	72.82	9.47	3	96.42	11.48	3	69.53	16.30	3	98.86	11.47
	4 ng/mL	3	60.79	9.06	3	94.31	8.69	3	50.92	11.05	3	96.27	10.24
	8 ng/mL	3	50.83	8.17	3	94.95	7.68	3	39.28	10.56	3	101.91	12.97
	16 ng/mL	3	43.98	11.37	3	94.89	12.90	3	29.95	9.25	3	101.74	13.21
	32 ng/mL	3	37.26	10.90	3	96.52	3.74	3	23.97	7.65	3	98.00	11.39
	64 ng/mL	3	30.63	8.96	3	96.98	12.86	3	20.51	6.56	3	100.44	12.96
	128 ng/mL	3	24.54	6.01	3	96.88	10.71	3	18.02	5.24	3	94.25	14.70
	256 ng/mL	3	21.06	3.99	3	89.85	10.80	3	16.37	4.37	3	95.29	5.53
SW48	0 ng/mL	3	100.00	---	3	100.31	1.90	3	100.00	---	3	99.87	2.09
	2 ng/mL	3	89.23	6.77	3	98.25	3.72	3	75.83	16.90	3	98.05	3.62
	4 ng/mL	3	73.50	7.68	3	98.64	2.13	3	56.86	10.79	3	97.82	4.66
	8 ng/mL	3	57.51	9.37	3	98.21	3.44	3	40.00	9.65	3	98.33	3.88
	16 ng/mL	3	49.86	9.84	3	98.19	5.00	3	30.79	7.91	3	99.24	4.06
	32 ng/mL	3	43.14	7.92	3	98.23	2.90	3	24.65	6.79	3	96.37	6.41
	64 ng/mL	3	39.72	8.27	3	96.86	6.92	3	22.17	5.93	3	95.22	5.86
	128 ng/mL	3	35.43	7.78	3	97.27	6.43	3	20.16	5.02	3	96.17	6.95
	256 ng/mL	3	32.70	7.83	3	97.04	4.62	3	18.03	4.44	3	98.13	5.85

Supplementary Table 5 (to Figure 5A-D)

			control			2.5 µM ASTX660			2.5 µM Birinapant		
		KillerTRAIL	n	mean (%)	SD (%)	n	mean (%)	SD (%)	n	mean (%)	SD (%)
HCT116	wt	0 ng/mL	3	100.00	---	3	95.91	7.47	3	94.91	2.78
		2 ng/mL	3	81.52	8.92	3	61.69	11.33	3	51.18	14.72
		4 ng/mL	3	74.97	9.97	3	52.67	10.62	3	38.05	12.79
		8 ng/mL	3	61.61	13.00	3	43.39	9.86	3	28.35	10.55
		16 ng/mL	3	45.35	13.78	3	33.08	8.69	3	20.11	8.07
		32 ng/mL	3	34.44	9.84	3	28.98	8.14	3	17.35	6.51
		64 ng/mL	3	28.67	7.86	3	25.27	6.80	3	15.77	6.13
		128 ng/mL	3	25.25	7.25	3	22.31	6.66	3	14.60	5.50
		256 ng/mL	3	25.38	6.92	3	22.41	6.12	3	14.48	5.60
	Casp8 KO	0 ng/mL	3	100.00	---	3	100.59	0.65	3	96.99	3.32
		2 ng/mL	3	101.39	4.79	3	97.84	6.86	3	91.94	10.60
		4 ng/mL	3	102.50	3.67	3	102.92	8.75	3	101.88	11.07
		8 ng/mL	3	102.52	1.19	3	102.09	9.66	3	97.80	9.89
		16 ng/mL	3	102.94	5.66	3	103.72	10.08	3	97.30	9.39
		32 ng/mL	3	104.24	5.88	3	101.99	8.25	3	97.82	5.88
		64 ng/mL	3	101.15	5.45	3	106.27	5.77	3	100.99	6.99
		128 ng/mL	3	102.57	3.13	3	102.60	10.00	3	100.25	11.17
		256 ng/mL	3	106.85	5.65	3	103.78	10.17	3	95.32	2.63
	BID KO	0 ng/mL	3	100.00	---	3	99.73	1.87	3	97.15	4.28
		2 ng/mL	3	106.25	4.24	3	103.18	11.00	3	93.55	8.01
		4 ng/mL	3	100.87	3.23	3	102.13	12.70	3	89.19	6.80
		8 ng/mL	3	102.54	5.16	3	100.29	8.97	3	80.77	3.70
		16 ng/mL	3	100.58	3.49	3	96.09	6.88	3	72.97	3.10
		32 ng/mL	3	95.05	1.45	3	97.81	8.65	3	67.05	2.18
		64 ng/mL	3	94.10	2.34	3	95.01	7.48	3	61.57	3.31
		128 ng/mL	3	95.09	2.28	3	90.71	5.65	3	56.60	5.03
		256 ng/mL	3	97.37	4.11	3	92.44	2.20	3	54.20	4.94
	BID KO + BID D60E	0 ng/mL	3	100.00	---	3	100.30	3.13	3	96.20	2.65
		2 ng/mL	3	102.59	5.75	3	101.69	10.65	3	90.57	7.19
		4 ng/mL	3	98.94	4.45	3	99.31	13.06	3	86.00	7.29
		8 ng/mL	3	99.27	5.95	3	97.29	15.19	3	79.34	5.31
		16 ng/mL	3	95.99	6.55	3	93.92	10.82	3	74.80	4.23
		32 ng/mL	3	95.03	7.99	3	92.07	16.22	3	69.85	4.34
		64 ng/mL	3	92.73	10.44	3	88.81	11.73	3	64.10	3.09
		128 ng/mL	3	92.70	6.41	3	83.53	12.80	3	59.59	4.01
		256 ng/mL	3	94.33	7.06	3	90.00	10.06	3	56.30	3.51

Supplementary Table 5 (continued, to Figure 5A-D)

			**control**			**2.5 µM ASTX660**			**2.5 µM Birinapant**		
		KillerTRAIL	n	mean (%)	SD (%)	n	mean (%)	SD (%)	n	mean (%)	SD (%)
**HCT116**	**BID KO + BID G94E**	0 ng/mL	3	100.00	---	3	100.43	4.07	3	96.43	4.19
		2 ng/mL	3	99.06	6.63	3	97.63	10.62	3	87.78	6.47
		4 ng/mL	3	100.83	8.96	3	96.98	13.75	3	84.23	6.62
		8 ng/mL	3	98.03	7.34	3	94.13	13.08	3	78.91	5.84
		16 ng/mL	3	95.38	6.10	3	92.73	12.75	3	73.55	3.16
		32 ng/mL	3	94.31	8.90	3	92.61	15.02	3	69.53	2.84
		64 ng/mL	3	90.15	8.72	3	91.17	15.26	3	65.59	2.52
		128 ng/mL	3	90.68	7.97	3	88.81	13.91	3	60.75	3.25
		256 ng/mL	3	94.79	5.68	3	88.60	11.86	3	58.38	3.44
	**BAX/BAK DKO**	0 ng/mL	3	100.00	---	3	94.37	7.54	3	93.44	4.23
		2 ng/mL	3	95.47	8.45	3	85.52	9.50	3	71.42	4.64
		4 ng/mL	3	91.18	8.78	3	80.11	10.92	3	71.03	4.67
		8 ng/mL	3	90.12	8.88	3	79.74	7.03	3	68.01	3.63
		16 ng/mL	3	88.20	8.12	3	79.98	7.34	3	65.36	2.46
		32 ng/mL	3	84.00	7.79	3	77.52	9.36	3	64.89	6.36
		64 ng/mL	3	82.28	5.82	3	77.05	6.32	3	62.06	3.61
		128 ng/mL	3	82.87	5.30	3	75.11	8.24	3	60.53	4.98
		256 ng/mL	3	84.06	3.29	3	76.70	5.46	3	61.38	3.09
**DLD1**	**wt**	0 ng/mL	3	100.00	---	3	101.15	3.89	3	99.51	8.57
		2 ng/mL	3	79.79	10.42	3	62.61	4.84	3	58.55	9.15
		4 ng/mL	3	72.17	7.05	3	52.42	2.76	3	47.82	7.37
		8 ng/mL	3	57.91	4.07	3	37.70	4.20	3	35.59	8.30
		16 ng/mL	3	41.11	5.42	3	24.81	5.14	3	23.67	9.45
		32 ng/mL	3	28.13	5.20	3	18.96	4.54	3	16.83	6.55
		64 ng/mL	3	20.23	3.78	3	15.19	5.21	3	14.74	6.03
		128 ng/mL	3	15.62	4.79	3	13.54	5.14	3	13.36	5.99
		256 ng/mL	3	16.05	4.90	3	13.81	5.27	3	14.32	6.16
	**BAX/BAK KO**	0 ng/mL	3	100.00	---	3	94.26	3.25	3	95.94	7.01
		2 ng/mL	3	90.78	2.38	3	88.70	1.83	3	85.70	4.47
		4 ng/mL	3	90.22	2.61	3	84.72	0.41	3	80.43	1.62
		8 ng/mL	3	89.05	2.29	3	86.18	5.82	3	74.32	3.39
		16 ng/mL	3	84.81	6.78	3	76.05	2.21	3	56.85	1.73
		32 ng/mL	3	67.90	2.54	3	64.32	5.42	3	37.22	7.14
		64 ng/mL	3	49.05	1.44	3	50.94	4.73	3	29.72	6.92
		128 ng/mL	3	39.24	4.79	3	40.54	7.33	3	24.74	6.14
		256 ng/mL	3	32.52	7.20	3	35.51	7.61	3	24.53	7.00

Supplementary Table 5 (continued, to Figure 5A-D)

			**control**			**2.5 µM ASTX660**			**2.5 µM Birinapant**		
		KillerTRAIL	n	mean (%)	SD (%)	n	mean (%)	SD (%)	n	mean (%)	SD (%)
**SW48**	**wt**	0 ng/mL	3	100.00	---	3	98.47	4.88	3	99.31	4.69
		2 ng/mL	3	96.43	1.68	3	65.33	11.07	3	44.55	15.00
		4 ng/mL	3	89.93	4.62	3	51.81	10.91	3	31.09	10.19
		8 ng/mL	3	73.58	8.97	3	43.91	8.86	3	25.69	8.00
		16 ng/mL	3	56.54	8.75	3	37.88	8.73	3	21.79	7.95
		32 ng/mL	3	48.68	9.22	3	34.30	9.37	3	19.52	7.88
		64 ng/mL	3	43.80	8.27	3	31.61	8.95	3	17.61	7.67
		128 ng/mL	3	40.18	7.53	3	28.27	8.82	3	15.68	6.82
		256 ng/mL	3	39.08	7.62	3	27.82	8.62	3	15.75	6.49
	**BAX/BAK DKO**	0 ng/mL	3	100.00	---	3	94.83	1.89	3	74.67	4.98
		2 ng/mL	3	101.25	1.48	3	82.84	5.21	3	49.84	2.04
		4 ng/mL	3	100.42	4.40	3	81.12	4.83	3	44.27	1.92
		8 ng/mL	3	99.22	5.30	3	80.27	4.71	3	39.74	1.85
		16 ng/mL	3	99.06	4.77	3	79.09	3.26	3	35.47	2.66
		32 ng/mL	3	96.22	4.65	3	76.05	3.42	3	32.99	2.65
		64 ng/mL	3	92.75	5.64	3	72.77	1.71	3	31.33	2.59
		128 ng/mL	3	91.40	2.69	3	70.08	1.18	3	28.82	2.32
		256 ng/mL	3	92.29	1.23	3	64.84	9.35	3	29.59	3.72
**HCT116**	**BIM KO**	0 ng/mL	3	100.00	---	3	96.95	5.80	3	97.46	3.35
		2 ng/mL	3	88.83	7.58	3	71.66	3.08	3	54.55	3.01
		4 ng/mL	3	85.19	6.75	3	60.32	1.78	3	35.48	4.02
		8 ng/mL	3	77.15	4.57	3	41.70	6.42	3	24.42	5.13
		16 ng/mL	3	63.34	3.32	3	29.26	6.56	3	20.06	4.95
		32 ng/mL	3	41.39	7.87	3	23.06	5.12	3	18.84	5.49
		64 ng/mL	3	25.74	6.21	3	22.29	6.42	3	17.90	5.66
		128 ng/mL	3	22.15	6.31	3	21.10	6.26	3	17.01	5.54
		256 ng/mL	3	21.90	6.03	3	21.75	6.52	3	17.44	5.42

Supplementary Table 6 (to Figure 3A)

	FLAG-TWEAK + anti-FLAG	control			5µM ASTX660		
		n	mean (%)	SD (%)	n	mean (%)	SD (%)
**DLD1**	0 ng/mL	3	100	---	3	94.21	0.95
	50 ng/mL	3	96.94	2.98	3	93.50	2.80
	100 ng/mL	3	95.97	0.97	3	94.22	4.03
	200 ng/mL	3	97.19	6.23	3	91.57	0.13
	400 ng/mL	3	99.79	2.07	3	98.51	0.40
**HCT8**	0 ng/mL	3	100	---	3	99.25	6.17
	50 ng/mL	3	97.53	3.82	3	92.07	4.83
	100 ng/mL	3	100.94	6.06	3	96.04	1.46
	200 ng/mL	3	99.32	9.24	3	90.64	1.44
	400 ng/mL	3	104.70	9.02	3	100.23	2.03
**HCT116**	0 ng/mL	3	100	---	3	94.88	1.99
	50 ng/mL	3	93.09	2.96	3	93.28	5.11
	100 ng/mL	3	92.41	1.84	3	98.02	10.25
	200 ng/mL	3	96.41	3.78	3	89.20	4.00
	400 ng/mL	3	99.70	1.16	3	98.52	2.88
**SW48**	0 ng/mL	3	100	---	3	92.94	2.68
	50 ng/mL	3	100.64	1.85	3	93.09	4.68
	100 ng/mL	3	101.54	0.91	3	94.19	1.85
	200 ng/mL	3	101.83	0.67	3	93.67	4.19
	400 ng/mL	3	98.98	0.18	3	93.49	4.75

Supplementary Table 7 (to Figure 3B)

		5µM ASTX660			5µM Birinapant		
		n	mean (%)	SD (%)	n	mean (%)	SD (%)
**DLD1**	ctr	3	95.80	5.69	3	97.60	4.70
	2µM ABT-737	3	92.93	3.57	3	87.67	3.43
	2µM A1331852	3	92.51	13.06	3	83.36	6.15
	2µM S63845	3	99.21	7.62	3	96.75	6.78
**HCT8**	ctr	3	106.09	6.60	3	98.17	4.03
	2µM ABT-737	3	103.13	3.52	3	94.22	2.70
	2µM A1331852	3	93.67	5.77	3	90.16	2.67
	2µM S63845	3	98.22	2.24	3	88.65	1.13
**HCT116**	ctr	3	99.08	5.33	3	93.58	2.26
	2µM ABT-737	3	94.80	10.39	3	82.41	4.19
	2µM A1331852	3	76.25	5.23	3	75.39	5.01
	2µM S63845	3	92.91	9.23	3	84.34	7.26
**SW48**	ctr	3	81.09	6.77	3	78.31	3.12
	2µM ABT-737	3	74.01	6.08	3	70.44	4.27
	2µM A1331852	3	67.18	4.85	3	63.68	2.77
	2µM S63845	3	88.96	11.19	3	94.01	8.93
